# From ISO 9001:2015 to ISO 20387:2018: a proposed implementation roadmap for biobanks

**DOI:** 10.3389/fgene.2026.1953941

**Published:** 2026-09-30

**Authors:** Michael Zuniga, Denis Marino, Gabriele Hartl, Berthold Huppertz, Zisis Kozlakidis, Karine Sargsyan

**Affiliations:** 1 MSc in Biobanking, Medical University of Graz, Graz, Austria; 2 OncoBiobank Shared Resource, Cancer Center, Cedars-Sinai Medical Center, Los Angeles, CA, United States; 3 International Biobanking and Education, Medical University of Graz, Graz, Austria; 4 Division of Cell Biology, Histology and Embryology, Gottfried Schatz Research Center, Medical University of Graz, Graz, Austria; 5 International Agency for Research and Cancer, World Health Organization, Lyon, France; 6 F. Widjaja Inflammatory Bowel Institute, Cedars-Sinai MIRIAD IBD Biobank, Los Angeles, CA, United States; 7 Department of Digital Medicine and Artificial Intelligence, Yerevan State Medical University, Yerevan, Armenia

**Keywords:** accreditation, biobank best practice, biobank implementation guideline, biobank management, international standards, quality management system, standardization in biobanking

## Abstract

**Introduction:**

Biobanks play an important role in biomedical research because of the availability of vast quantities of high-quality biological specimens and related data. However, biobank-specific standards such as ISO 20387:2018 have been infrequently implemented. This study aimed to develop a practical implementation roadmap to assist ISO 9001:2015-certified biobanks in adopting ISO 20387:2018.

**Methods:**

The implementation roadmap was developed through a scientific literature review and a comparative, clause-level analysis (crosswalk) of ISO 9001:2015 and ISO 20387:2018. The analysis identified areas of overlap between the standards and requirements specific to ISO 20387:2018.

**Results:**

The crosswalk demonstrated substantial overlap between the two standards while identifying additional ISO 20387:2018 requirements related to financial viability assessment, plans for future expansion, additional specimen-collection standards, authentication protocols, and reporting requirements. Based on these findings, we developed a practical, time-bound implementation framework that leverages existing ISO 9001:2015 systems to help structure the transition to ISO 20387:2018. The proposed roadmap can be completed within an estimated 17-20 weeks.

**Discussion/Conclusion:**

The implementation framework provides a structured approach for ISO 9001:2015-certified biobanks seeking to adopt ISO 20387:2018 and may help make accreditation more attainable for resource-constrained biobanks. We identify testable assumptions about feasibility, duration, and organizational benefits, and highlight the need for formal training in biobank management to sustain implementation. Prospective validation is needed to determine whether the proposed timeline and anticipated organizational benefits are achieved in practice.

## 1 Introduction

In the era of big data, biobanks play an important role in providing high-quality biological samples and associated data that enable in-depth analysis and thorough integration in future biomedical research, clinical practice, and public health (Sun et al., 2025). Biospecimens serve as a critical bridge between clinical information and the evolving field of precision medicine, which aims to prevent and treat diseases by adapting treatment based on individual genetics, clinical, environmental, and lifestyle factors (Strianese et al., 2020). As recently demonstrated by Osipov et al. (2024), integrating multi-omics data can improve outcome prediction in patients with pancreatic adenocarcinoma.

Supporting the growing field of biomedical research inherently involves harmonizing biobanking processes, including data collection, processing, conservation, distribution, and management of biological samples (Liu and Pollard, 2015). However, heterogeneity in pre-analytical handling, storage, and data documentation contributes to poor reproducibility and undermines the value of biobanks for translational research (Riegman et al., 2019).

Reproducibility problems in preclinical studies have substantial economic and social implications. Recent surveys indicate that 40%–87% of scientists have experienced failure to reproduce results in their own or others’ experiments, and 72% of biomedical researchers acknowledge a reproducibility crisis (Baker, 2016; Cobey et al., 2024). Although there is currently no global consensus on biobanking activities and associated regulations, biobank networks and governmental or international institutions are leading initiatives to establish Quality Management Systems (QMS) that cover the collection, long-term storage, retrieval, and distribution of biospecimens and associated data.

Despite these initiatives, fragmentation across regulatory frameworks and standards creates implementation challenges (Schmid and Bledsoe, 2025). Credible references include best practice guidelines from the International Society for Biological and Environmental Repositories (ISBER) (International Society for Biological and Environmental Repositories ISBER, 2023), the Biorepositories and Biospecimen Research Branch (BBRB) of the National Institute of Health (NIH) - National Cancer Institute (NCI) (National Cancer Institute NCI, 2016), the European Commission, Biological and Biomolecular Resources Research Infrastructures (BBMRI‐ERIC) (Council of Europe: Committee of Ministers, 2016), and the International Agency for Research on Cancer (IARC) (Mendy et al., 2017). Certification and accreditation initiatives, such as the Biorepository Accreditation Program (BAP) from the College of American Pathologists (CAP) (College of American Pathologists CAP, 2020), the CTRNet Biobank Certification Program (Biobank Resource Centre, 2020), and the Healthcare Improvement Scotland Accreditation of NHS Boards’ Human Tissue Collection for Research (NHS Research Scotland, 2016), help ensure quality, transparency, reproducibility, and effective specimen and data management.

According to Leelatian et al. (2026), 104 biobanks in the United States are fully CAP BAP-accredited. This program has been in place for 13 years and is designed to minimize preanalytical variability, which is an essential aspect of translational research. While these programs and guidelines have improved the quality of individual biobanks, their scope, legal status, and geographical coverage vary, contributing to a fragmented standardization landscape.

In response to multiple biobanking guidelines, the International Organization for Standardization (ISO) developed ISO 20387:2018 (International Organization for Standardization, 2018), the first international standard specifically for biobanks. It demonstrates the technical competence, impartiality, and consistent operation of biobanks by addressing quality management, personnel competence, infrastructure, and ethical and legal governance (International Organization for Standardization, 2018).

Seven years after the release of ISO 20387:2018, most biobanks still hold ISO 9001:2015 or ISO 17025:2017 certifications. According to the BBMRI-ERIC Directory, accessed in January 2026, among the 406 registered biobanks, only 50 (12.3%) have any form of certification: 3 (0.7%) hold ISO 15189, 1 (0.2%) hold ISO 17025, 2 (0.5%) hold both ISO 17025 and ISO 15189, and 28 (6.9%) are certified under ISO 9001. Furthermore, 11 (2.7%) are accredited under ISO 20387, 4 (1.0%) have both ISO 20387 and ISO 9001, and 1 (0.2%) possesses both ISO 17025 and ISO 20387 (BBMRI-ERIC, 2026a).

Existing publications provide general implementation guidance for ISO 20387:2018 and case reports of accreditation journeys, but they do not offer a structured roadmap specifically tailored to biobanks already certified under ISO 9001:2015. We therefore aimed to develop and describe a clause-level crosswalk and implementation roadmap that (i) quantifies the overlap between ISO 9001:2015 and ISO 20387:2018, (ii) identifies ISO 20387-specific gaps, and (iii) translates these into a practical, time-bounded, role-based implementation guide for ISO 9001:2015-certified biobanks.

The guide aims to support biobanks that seek ISO 20387:2018 accreditation, anticipating future demand for standardized biobank processes from stakeholders, including researchers, government agencies, and private companies. We argue that clause-level crosswalk can be used to concentrate implementation effort on ISO 20387:2018-specific gaps and, when combined with a structure governance model, yields a feasible, time-bounded roadmap for ISO 9001:2015-certified biobanks.

## 2 Materials and methods

### 2.1 Study setting and scope

This study explores the feasibility of implementing ISO 20387:2018 in biobanks already certified under ISO 9001:2015 for the reception, processing, analysis, transport, distribution, and storage of biological materials and associated data. We developed the implementation roadmap within a single organizational setting that had an established ISO 9001:2015-certified quality management system and multidisciplinary institutional support for quality, technical, administrative, and operational activities. This setting provided the basis for defining the implementation of activities, governance structure, and allocation of responsibilities presented in the roadmap.

### 2.2 Narrative literature review

We searched three electronic databases (PubMed/MEDLINE, Scopus, and Web of Science Core Collection) for records published between January 2015 to December 2025. Search strategies combined controlled vocabulary (where available) and free-text terms within three concept blocks using the Boolean operator AND: (1) biobanking terms (e.g., “biobank” “biorepository”); (2) Standardization terms (e.g., “Standardization”, “Implementation”); and (3) ISO terms (e.g., “ISO 9001”, “ISO 20387”). Search strategies were adapted to the syntax and indexing of each database.

Grey literature, policy documents, and regulatory instruments were identified through targeted hand-searching of the websites and document repositories of organizations including the International Society for Biological and Environmental Repositories (ISBER), the National Cancer Institute (NCI), WHO International Agency for Research on Cancer (IARC), Organization for Economic Cooperation and Development (OECD), and the International Organization for Standardization (ISO). For each organization, we focused on sections and repositories dedicated to standards, guidance, or policy relevant to biobanking and quality management. Reference lists of included records and key review articles were screened for additional sources.

### 2.3 Clause-level crosswalk of ISO 9001:2015 and ISO 20387:2018

A comparative crosswalk analysis was conducted to determine the extent to which the requirements of ISO 20387:2018 are addressed by corresponding requirements in ISO 9001:2015. The analysis was conducted at the clause and subclause level, using ISO 20387:2018 as the reference standard.

For every ISO 20387:2018 requirement statement, we examined ISO 9001:2015 to identify one or more clauses or subclauses that could reasonably be interpreted as addressing that requirement. When such correspondence was identified, the relevant ISO 9001:2015 clause number(s) were recorded in the crosswalk and that ISO 20387:2018 requirement was coded as “addressed” for the purposes of the comparative analysis. When no corresponding ISO 9001:2015 requirement could be identified, the requirement was coded as “not addressed,” indicating an identified gap requiring additional consideration during implementation.

The analysis applied a binary classification at the level of individual ISO 20387:2018 requirement statements, based on the presence (“addressed”) or absence (“not addressed”) of at least one corresponding ISO 9001:2015 clause. This classification was applied consistently across all ISO 20387:2018 clauses and subclauses assessed. The resulting mapping was used to quantify the proportion of ISO 20387:2018 requirements addressed by ISO 9001:2015 and to identify areas requiring additional implementation activities.

Percentages of ISO 20387:2018 requirements addressed by ISO 9001:2015 were calculated as the proportion of ISO 20387:2018 clauses or subclauses for which a corresponding ISO 9001:2015 clause or subclause was identified, divided by total number of ISO 20387:2018 clauses or subclauses assessed within that section multiplied by 100.

### 2.4 Development of the implementation roadmap

We combined a narrative literature review with a clause-level crosswalk analysis of ISO 9001:2015 and ISO 20387:2018 and used the integrated findings to develop a practical implementation guide for ISO 20387:2018 in ISO 9001:2015–certified biobanks. For each ISO 20387:2018 clause, we identified the corresponding ISO 9001:2015 clauses and existing processes in the host biobank and reported implementation barriers, facilitators, and best practices from the literature. We then mapped these onto concrete implementation activities, responsible roles, and estimated timelines.

## 3 Results

### 3.1 Study selection and characteristics

A total of 240 articles were identified through searches conducted in PubMed/MEDLINE (n = 55), Scopus (n = 81), and Web of Science Core Collection (n = 104). After removal of 34 duplicate records, 206 publications were scanned based on title and abstract. Of these, 187 records were excluded because they did not address the scope of the review. Nineteen publications were subsequently sought for full-text assessment; one article could not be retrieved, and 18 full-text articles were assessed for eligibility. Following full-text assessment, one additional article was excluded because it did not provide evidence related to implementation of ISO 9001:2015 or ISO 20387:2018, nor did it include a comparative analysis of the two standards. Finally, 17 publications were retained. These publications addressed topics including biobank quality management, standardization of biobanking processes, implementation of quality management systems, certification approaches, and requirements associated with ISO 9001:2015 and ISO 20387:2018 (see Figure 1; Table 1).

**FIGURE 1:**
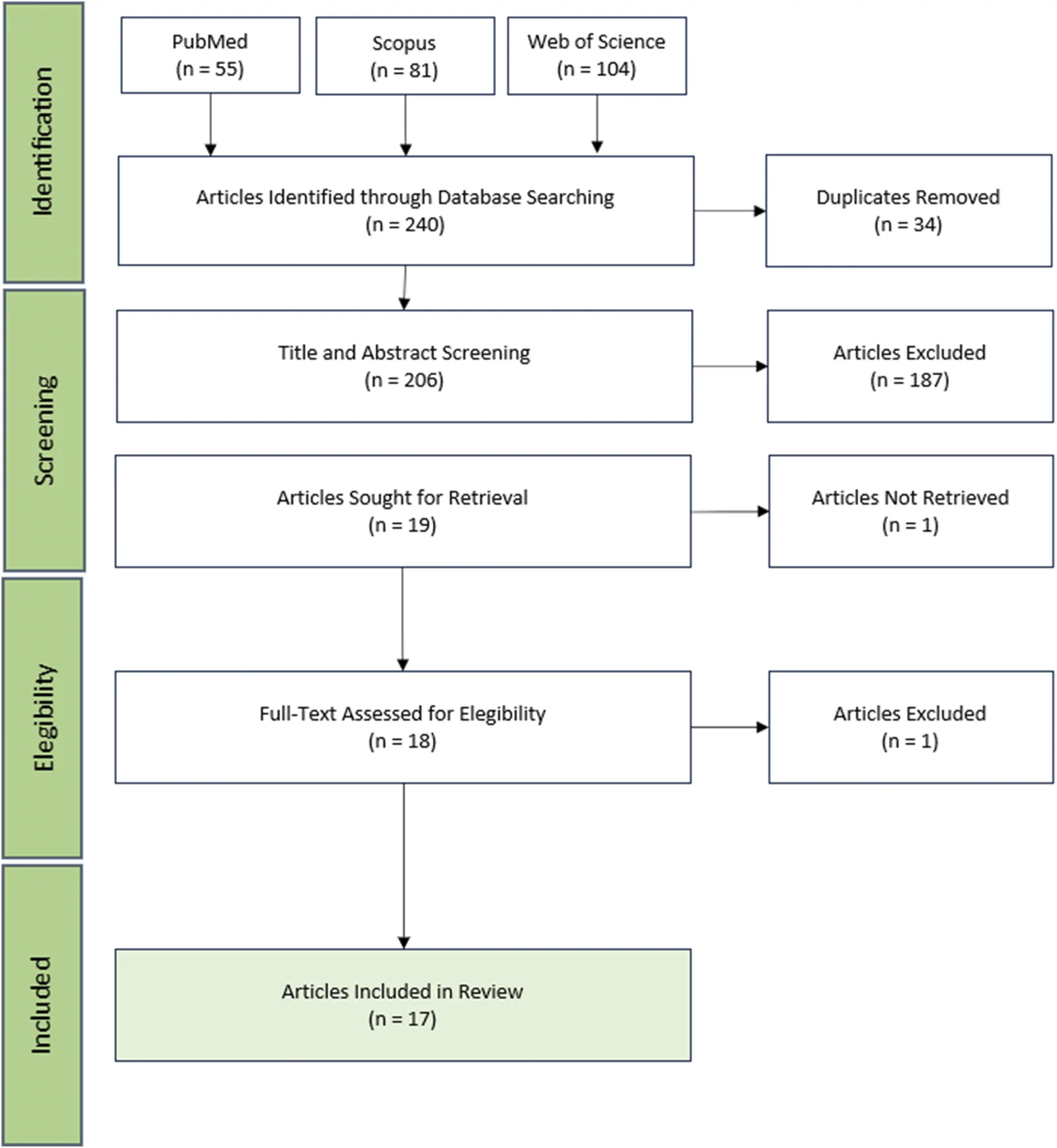
PRISMA 2020 flow chart for narrative literature search and selection process.

**TABLE 1:** Characteristics of the 17 included studies.

Authors (year)	Study design	Key findings
ISO 9001:2015		
Pereira et al. (2015)	Narrative review/regulatory analysis	Reviews quality-management and laboratory standards applicable to blood screening laboratories and highlights the need to complement general quality-management requirements with laboratory-specific technical and operational requirements needed beyond ISO 9001.
Calleros-Basilio et al. (2016)	Quantitative quality-control study	Demonstrates the importance of systematic quality control and quality assurance for biological materials, including assessment of sample quality, process performance, and fitness for purpose.
Corsi et al. (2020)	Experience report	Describes the implementation of an ISO 9001:2015-based quality management system in a transplantation tissue bank over a 13-month period, illustrating the application of structured quality-management processes in tissue banking.
Laun et al. (2022)	Retrospective uncontrolled before-and-after comparison	Reports measurable improvements in operational outcomes following implementation of an ISO 9001-based quality management system in a cornea eye bank.
ISO 20387:2018		
Meinung et al. (2019)	Position piece/policy mapping	Describes development of ISO 20387 and discusses its role as a conformity-assessment standard for biobanks, emphasizing accreditation as a mechanism for monitoring biobank performance and demonstrating conformity.
Borisov et al. (2020)	Regulatory analysis and policy mapping	Describes ISO 20387 as a management-system standard addressing competence, quality management, biosafety and infrastructure, and emphasizes that the standard provides a framework adaptable to individual biobank conditions rather than prescribing specific storage or processing parameters.
Annaratone et al. (2021)	Narrative review	Identifies standardization, quality management, infrastructure, personnel competence, documentation, and governance as key components of reliable biobank operations.
Corradi et al. (2025)	Legal and regulatory analysis	Describes ISO 20387 requirements across general, structural, resource, process and quality management domains, emphasizing that ISO 9001 certification alone does not establish biobank-specific competence.
Tarling et al. (2020)	Comparative document and regulatory analysis	Compares 362 CTRNet and 322 ISO 20387 requirements and reclassifies them into 13 categories, demonstrating substantial areas of correspondence as well as biobank-specific differences between the two standards.
Mouttham et al. (2021)	Implementation case report	Provides a practical implementation case of ISO 20387 accreditation at a veterinary biobank. Including quality management system, processes definition, training personnel, documenting activities, internal review and preparing for external assessment.
Implementation of QMS		
Hannigan et al. (2019)	Implementation case report	Describes integration of ISO 9001 and ISO/IEC 27001 at Qatar Biobank, demonstrating how an existing an established quality management system can be integrated with additional management requirements.
Linsen et al. (2019)	Two cross-sectional surveys	Reports widespread use of formal quality-management systems among BBMRI.be biobanks, while identifying weaker implementation of areas such as impartiality statements and quality controls.
De Blasio and Biunno (2021)	Conceptual guidance article	Provides stepwise guidance for ISO 20387 implementation, from gap analysis, planning, documentation, implementation and auditing, and estimates timelines of 6–18 months and 2–4 person-months, in addition to accreditation and maintenance costs.
Carranza et al. (2026)	Review plus proposed guide with one implementation example	Proposes pathway from transitioning from laboratory sample store to a formal biobank using ISO 20387, highlighting the need to address technical, infrastructure, quality-management and operational requirements.
Governance, risk management and sustainability		
Yadav et al. (2020)	Survey-derived multi-case scenario analysis	Highlights the importance of risk management, emergency preparedness, operational continuity, and adaptable biobanking procedures for maintaining biobank actives under changing or disruptive conditions.
Rush et al. (2020)	Policy perspective with one bibliometric analysis	Proposes sustainability framework across inputs, internal processes and outputs, emphasizing performance measurement, stakeholder needs, resource plaining, and demonstration of biobank value.
De Palma et al. (2022)	Risk assessment case study	Applies a structured risk-assessment approach to a cancer biobank to identify and evaluate operational risks and support risk-reduction measures, highlighting risk assessment as an important component of biobank management within the ISO 20387 framework.

The results of the literature review, relevant standards, and international best-practice recommendations (e.g., ISBER, NCI, IARC, OECD, ISO) (International Society for Biological and Environmental Repositories ISBER, 2023; National Cancer Institute NCI, 2016; Mendy et al., 2017; International Organization fo r Standardization, 2018; Organization for Economic Co-operation and Development OECD, 2010; International Organization for Standardization, 2015) were used as supporting evidence to provide contextual and technical guidance for the proposed implementation approach.

### 3.2 Clause-level crosswalk of ISO 9001:2015 and ISO 20387:2018

The comparative analysis demonstrated substantial overlap between ISO 20387:2018 and ISO 9001:2015, particularly in areas related to quality management, organizational responsibilities, personnel competence, documented information, infrastructure, externally provided processes, process control, nonconformity outputs, monitoring, and continual improvement.

The crosswalk identified corresponding ISO 9001:2015 requirements for most of the ISO 20387:2018 provisions evaluated. However, several ISO 20387:2018 requirements were either only partially addressed by ISO 9001:2015 or had no direct corresponding requirement. Figure 2 shows that the vast majority of ISO 9001:2015 clauses map onto corresponding requirements within ISO 20387:2018, illustrating that ISO 20387 consolidates and extends ISO 9001.

**FIGURE 2:**
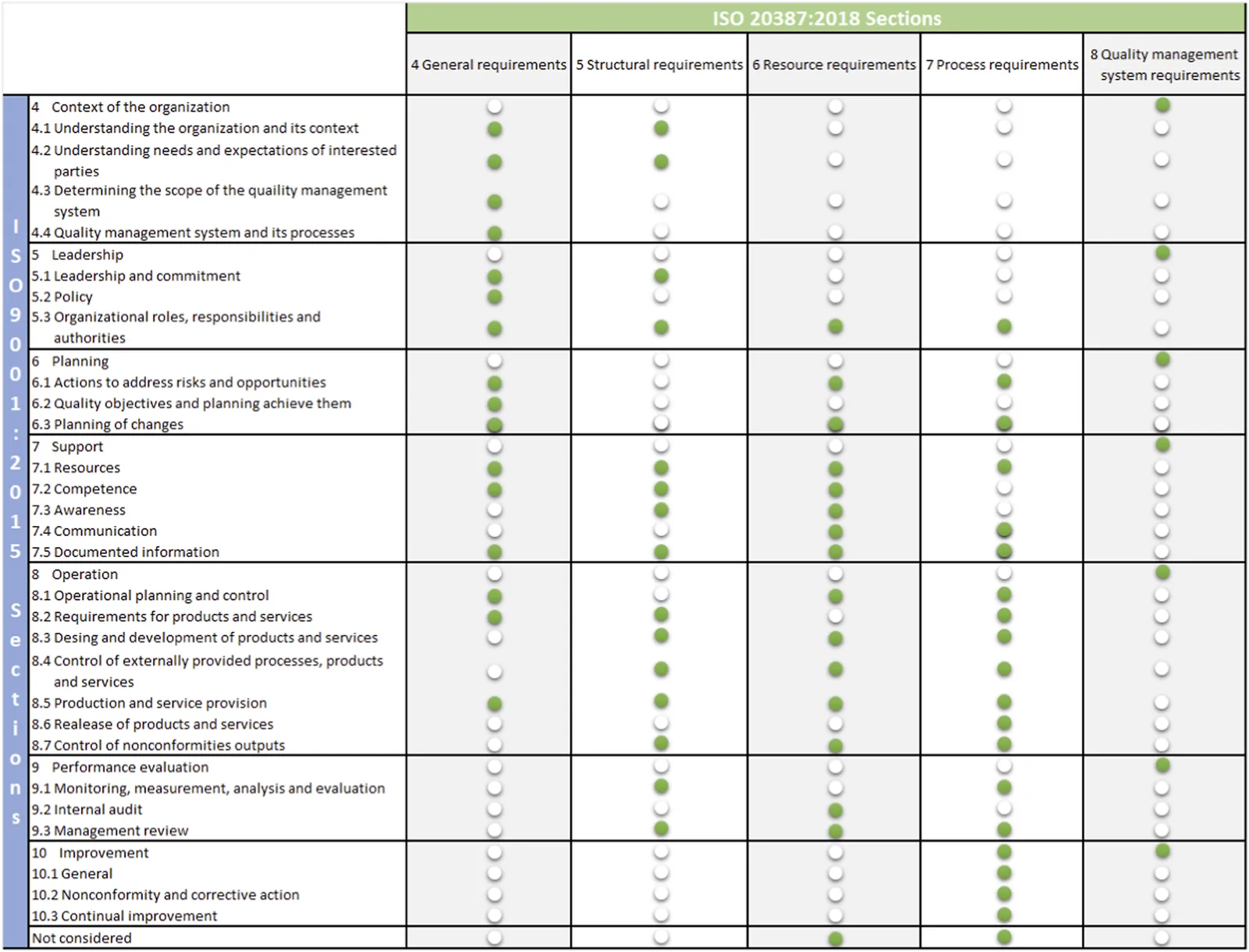
Comparison of requirement compliance between ISO 20387 and ISO9001.2015.

As shown in Figure 3, the alignment of these two standards is complementary. The analysis found full compliance (100%) with the general requirements (Section 4), structural requirements (Section 5), and quality management system requirements (Section 8). High compliance was also observed in Sections 6 and 7 (resource and process requirements) at 96% and 97%, respectively. A summary clause-by-clause crosswalk is provided in Supplementary Table S1.

**FIGURE 3:**
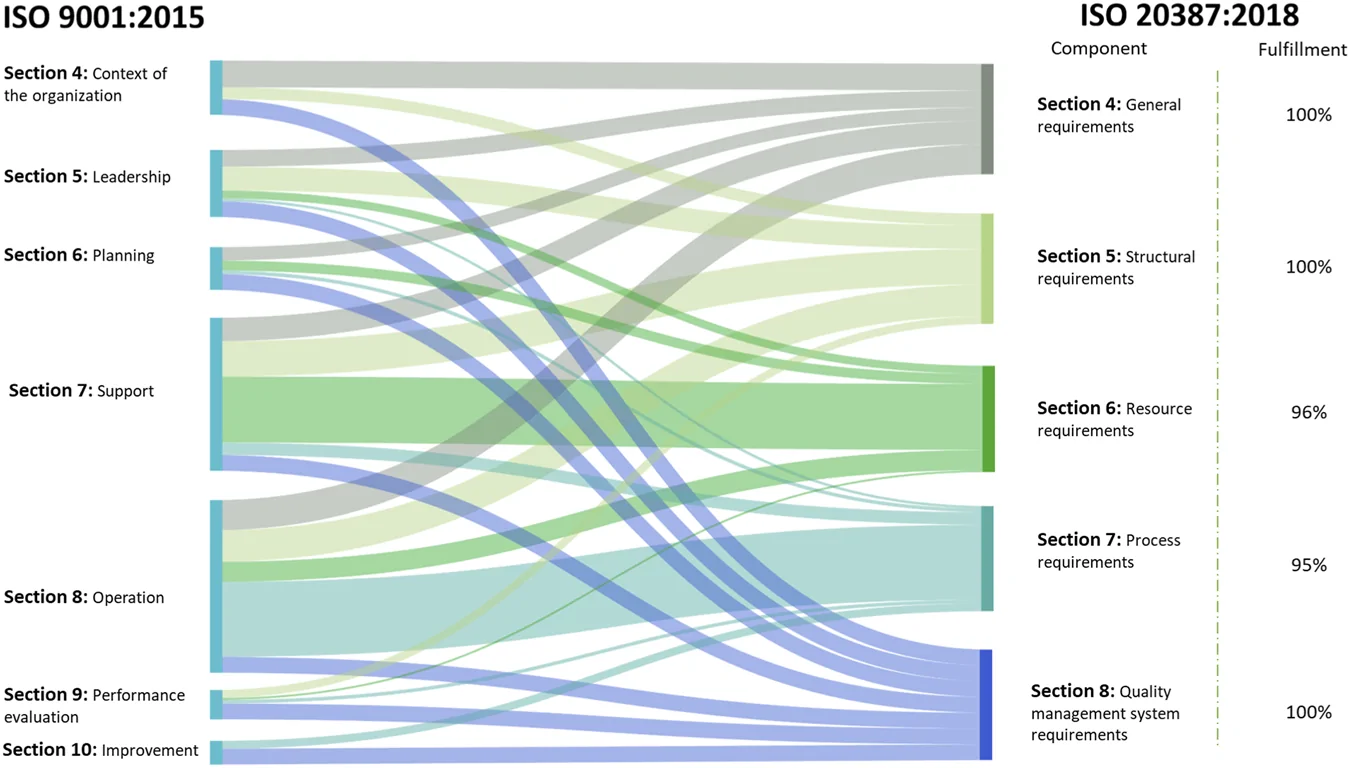
Sankey diagram showing the alignment of ISO 9001:2015 components with ISO 20387:2018 sections and corresponding percentage compliance for each section.

The gaps identified in Sections 6 and 7—financial viability assessments, facility planning, process requirements, data management, and reporting requirements—defined the core areas requiring additional implementation activities.

### 3.3 Development of the implementation roadmap

Based on the crosswalk analysis and a review of the relevant literature and standards, we synthesized a practical implementation roadmap for ISO 20387:2018 in ISO 9001:2015-certified biobanks. The roadmap is structured around an interdisciplinary workgroup and eight core task areas, each corresponding to one or more ISO 20387:2018 requirements that were not fully addressed by ISO 9001:2015 in our crosswalk (Sections 6 and 7).

These eight task areas are: (1) governance and interdisciplinary workgroup, (2) future expansion planning, (3) documentation and reporting requirements, (4) reception and authentication of biological materials, (5) pre-analytical workflows, (6) financial viability, (7) internal compliance review, and (8) conformity assessment.

The proposed implementation approach was developed through a structured, multi-task process encompassing technical, administrative, and organizational activities entailing several departments. Figure 4 presents the proposed implementation roadmap, including the key areas identified for the implementation readiness of ISO standard 20,387:2018 and the corresponding responsibility matrix. The matrix establishes the governance structure for each task, identifying the personnel responsible for execution, approval, decision-making, and consultative roles.

**FIGURE 4:**
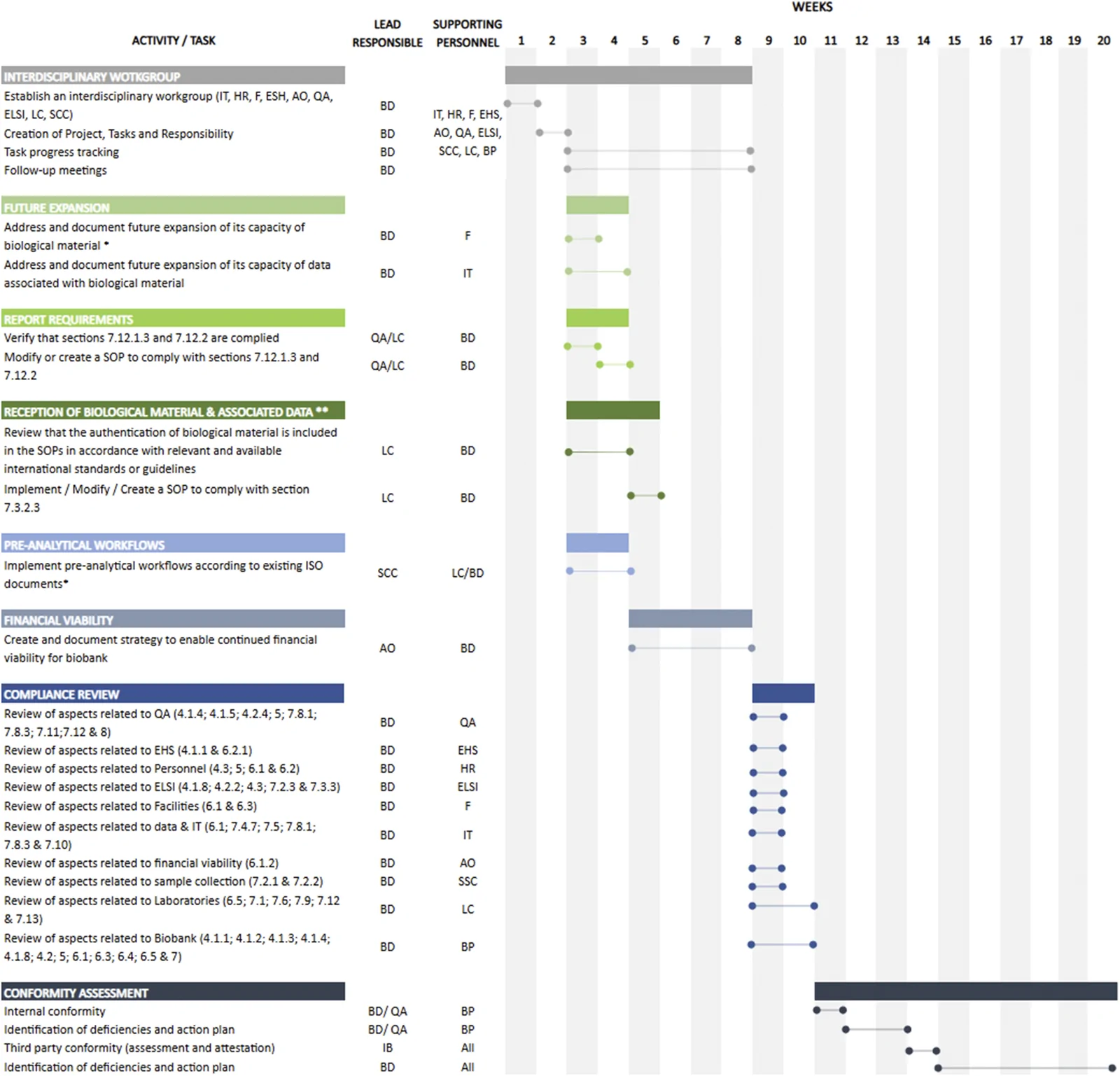
Gantt chart: Proposed roadmap for implementing ISO 20387:2018 in biobanks certified with ISO 9001:2015. BD, Biobank Director; IT, Information Technology Department; HR, Human Resources Department; F, Facilities Manager; EHS, Environmental Health and Safety; AO, Accounting Office Department; QA, Quality Assurance Manager; ELSI, Ethical, Legal, and Social Issues Department; SCC, Sample Collection Coordinator; LC, Laboratory Coordinator; BP, Biobank Personnel and IB, Impartial Body. * Not a mandatory requirement in the standard (should) ** When appropriate and applicable, depends on the type of biospecimens received.

#### 3.3.1 Interdisciplinary workgroup

Establishing an effective governance structure to coordinate the implementation of ISO 20387:2018 requires an interdisciplinary workgroup that represents the competencies relevant to the biobank’s scope of activities. In the roadmap proposed in this study, the workgroup is defined to include representatives from Information Technology Department (IT), Human Resources Department (HR), Facilities Manager (F), Environment Health and Safety Department (EHS), Accounting Office Department (AO), Quality Assurance Manager (QA), Ethics, Legal and Social Issues Department (ELSI), Sample Collection Coordinator (SCC), Laboratory Coordinator (LC), and Biobank Personnel (BP). However, this structure should not be interpreted as requiring a dedicated representative from each department. In smaller biobanks, for example, responsibilities may be combined within specialized expertise on a part-time or consultive basis. The size and composition of the implementation team should therefore be proportional to the biobank’s organizational structure, scope, operational complexity, and available resources.

As part of the proposed roadmap, we recommend that the interdisciplinary workgroup collaborate over an initial period of 8 weeks to coordinate and complete the implementation activities required for compliance with ISO 20387:2018 prior to the conformity assessment. Through organizational analysis, we identified the Biobank Director (BD) as the pivotal coordinator for implementation. Within this structure, the Biobank Director acted as the central coordinator, linking management, biobank staff, and participating departments by defining the project structure, assigning tasks and responsibilities, and organizing follow-up meetings.

#### 3.3.2 Future expansion

The requirement for future expansion is framed as a prospective planning exercise to support the biobank’s long-term operational sustainability. This exercise should include a five-to-ten-year forecast of the expected study portfolio, incorporating projections of the number and type of incoming studies, anticipated biospecimen accrual, expected sample use, and storage requirements after study closure. The analysis should also address the projected retention period for each collection, long-term sample storage, and custodial responsibilities. Additionally, future expansion planning includes assessing the necessary data storage capacity to preserve associated biospecimen data, including sample metadata, clinical and operational records, and data generated through future analyses and study results. The Biobank Director leads this requirement, with support and consultative input from the Facilities Manager and the Information Technology Department.

#### 3.3.3 Report requirements

This task covers the review and updating of standard operating procedures (SOPs) to include biological materials, in accordance with international standards and relevant guidelines. The Quality Assurance Manager and Laboratory Coordinator led this task, consulting with the Biobank Director. They should conduct a systematic review of the existing ISO 9001:2015 documentation to identify additional documents, procedures, or requiring creation or adaption to comply with ISO 20387. The review should cover standard operating procedures (SOPs), forms, flowcharts, manuals, external documents, and documents and records related to impartiality, confidentiality, organizational structure, personnel roles and responsibilities, training and competency of personnel, facilities, maintenance and calibration of equipment, and management of external providers. It also includes procedures for collecting, receiving, transporting, preparing, distributing, preserving, testing, analyzing, and storing biological materials. It also includes requirements for traceability, data management, control of nonconforming outputs, complaint handling, control of documents and records, identification of risks and opportunities, corrective actions, internal audits, and management review.

Because biobanks differ in their organizational structure, scope of operations, sample portfolio, and level of process complexity, each biobank should conduct a documentary review to ensure it adequately fulfills the general, structural, resource, and process requirements of ISO 20387, together with the requirements of the quality management system.

#### 3.3.4 Reception of BMaD (biological material and associated data)

This task applies only to biobanks that are required to authenticate biological materials (e.g., cell lines and microorganisms). The Laboratory Coordinator and Biobank Director established procedures to document authentication before these materials were accepted into the biobank. This task should define the categories of biological material requiring authentication, acceptance criteria, responsibilities, required documentation, and records to demonstrate compliance.

Authentication methods should be selected according to the type of biological material and current international recommendations (e.g., Short Tandem Repeat profiling for human cell lines and 16 S rRNA or ITS sequencing for microorganisms).

#### 3.3.5 Pre-analytical workflows

The Sample Collection Coordinator leads this task, and the Laboratory Coordinator and Biobank Director in supporting and consultative roles. This key task entails implementing workflows aligned with existing ISO standards to standardize biospecimen management and harmonize the collection, processing, and storage of biospecimens and associated data, thereby minimizing pre-analytical variability and preserving biospecimen quality for their intended use. Workflow implementation should be tailored to the type of biological material managed by each biobank and incorporate internationally recognized pre-analytical standards whenever applicable. For example, biobanks handling formalin-fixed paraffin-embedded (FFPE) tissue should implement the relevant parts of the ISO 20166 series for the isolation and analysis of DNA, RNA, and proteins from FFPE specimens, whereas ISO 20184:2018 and ISO 20186:2019 provide guidance for the pre-examination processes of frozen tissue and venous whole blood, respectively.

#### 3.3.6 Financial viability

The primary objective is to demonstrate that the biobank has the financial capacity to sustain its operations while maintaining the quality, integrity, and long-term preservation of biological materials and the associated data. The Accounting Office leads this task, and the Biobank Director serves as the approval and decision-making authority.

A financial sustainability plan should be developed based on a five-to ten-year forecast created in the Future Expansion task. The plan should estimate fixed costs, such as personnel, facility operation, equipment acquisition and depreciation, preventive maintenance, calibration, environmental monitoring, information technology infrastructure, software licensing, and quality management. It should also estimate the variable costs associated with specimen collection, processing, storage, distribution, consumables, and laboratory analyses.

To support long-term sustainability, a cost-recovery model that identifies the principal funding sources and mechanisms that support biobank operations is essential. Depending on the institutional context, these sources may include institutional funding, competitive research grants, sponsor-supported studies, fees for specimen processing or distribution, cost-recovery charges for storage services, inter-institutional service agreements, and collaborative research contracts.

The financial sustainability plan should incorporate operational indicators, such as annual operating costs, biospecimen processing and storage costs, storage occupancy rate, specimen utilization rate, percentage of cost recovery, annual maintenance and calibration expenditures, revenue generated through biobank services, and projected versus actual operational costs.

#### 3.3.7 Compliance review

The Compliance Review represents the final internal verification stage of the ISO 20387:2018 implementation project before proceeding to the conformity assessment stage. Coordinated by the Biobank Director and involving all functional areas engaged in the implementation, the review verifies that all activities, deliverables, and milestones defined during the interdisciplinary workgroup phase have been completed according to the assigned responsibilities and timeline.

The review should allow the implementation team to identify any remaining gaps, define additional actions if necessary, and confirm organizational readiness before moving on to the conformity assessment phase.

#### 3.3.8 Conformity assessment

This stage comprises two sequential phases: an internal conformity assessment conducted by biobank staff and the QA manager, followed by a third-party assessment conducted by an impartial accreditation body. The first phase evaluated all the processes aligned with the requirements of ISO 20387:2018 and identified areas requiring further attention. The subsequent phase would provide an independent evaluation of conformity with the applicable requirements. After each phase, deficiencies were identified, and corrective action plans should be implemented with the shared goal of improvement.

## 4 Discussion

Unlike previous case reports and conceptual guidance, which typically address ISO 20387:2018 implementation from a *de novo* perspective, our study starts from an existing ISO 9001:2015 QMS and provides a clause-level map and organizational model of how to reuse that infrastructure for ISO 20387 readiness.

### 4.1 Interpretation in context

This study reviewed and compared two ISO standards used for biobanks that differ in their scope and intended application. ISO 9001:2015 provides an operational foundation through the Quality Management System (QMS) standard. It is applicable to organizations of any type and size, with the objective of increasing customer satisfaction through improved process efficiency and consistent fulfillment of customer requirements (Carranza et al., 2026). On the other hand, ISO 20387:2018 is the international standard for biobanking quality and competence. It focuses on ensuring the impartiality, competence, and proper operation of biobank activities, with particular attention to the quality of biospecimens and associated data (International Organization for Standardization, 2018).

The implementation of these standards offers complementary benefits to biobanks. ISO 9001:2015 establishes a solid basis for a quality management system (QMS), whereas ISO 20387:2018 extends the intended application through specialized biobank-specific requirements that strengthen governance, ensure personnel competence, and promote sustainability (Wyderka et al., 2020).

### 4.2 Practical implications and external resources

To support this harmonized structure, ISO 20387:2018 explicitly references 25 ISO documents. These include ISO 9000 and ISO 9001 for quality management systems, ISO 15189:2012 for medical laboratory competence, and ISO 20166 (all parts) for the pre-examination processes of formalin-fixed, paraffin-embedded (FFPE) tissue. Other relevant standards include ISO/TS 20658 for sample handling, ISO 26000 for social responsibility, and ISO 27799 for health informatics and information security​3,434​. ISO 20387:2018 underscores the need to implement standardized pre-analytical ISO standards, such as those for isolating RNA/DNA/proteins from FFPE tissue, frozen tissue, or whole blood.

This layered structure indicates that ISO 20387:2018 consolidates and operationalizes existing standards rather than replacing them. From a practical perspective, this approach promotes the harmonization of diverse biobanking processes and expands quality requirements to include data management and governance, in addition to sample collection (International Organization for Standardization, 2020).

Our analysis suggests that, building on the overlap between ISO 9001:2015 and ISO 20387:2018, the internal preparation effort can be completed within an estimated minimum timeframe of 17–20 weeks, especially if the workgroup has previous experience with quality management systems and has a QMS in place. This estimated timeframe is shorter than the full accreditation timelines reported in similar case studies: a structured 12-month pathway at a public hospital biobank in Pisa (Zucca et al., 2026), and approximately 8 months at the Cornell Veterinary Biobank (Mouttham et al., 2021). These longer timelines reflect that the two repositories did not hold an ISO 9001:2015 certification or QMS before implementing ISO 20387:2018 (De Blasio and Biunno, 2021).

Similar to Davis et al. (2012) who performed a comparable crosswalk between CAP BAP and ISO 20387:2018 and found that the two standards differ substantially in the number and distribution of requirements (523 vs. 167), the present study shows that the implementation effort extends beyond the biobank itself. A quality management system involves multiple institutional departments that contribute to both core and supportive processes. To ensure continuity and effective management of the process, a dedicated role should be appointed to oversee and coordinate its entire implementation (De Blasio and Biunno, 2021). Prior experience with the certification process can expedite implementation by facilitating the establishment of an efficient QMS and ensuring compliance with regulatory requirements (Mouttham et al., 2021).

An important consideration in the proposed roadmap transition is that ISO 9001:2015-certified biobanks already have risk-based processes embedded within their QMS. The transition to ISO 20387:2018 does not introduce risk management as a novel quality activity. Instead, it involves a systematic evaluation of whether the current risk-management processes sufficiently address risks unique to biobanking. This risk management should be viewed as a cross-cutting element that supports the identification of gaps and the prioritization of actions throughout the proposed implementation roadmap (De Palma et al., 2022).

Biobanks preparing for ISO 20387:2018 accreditation are not limited to what they can do in-house. BBMRI-ERIC’s Quality Management Services provide partner biobanks access to self-assessment surveys, remote or on-site audits, and consultancy for QMS implementation. Biobanks that successfully complete the process receive a Quality Label listed in the BBMRI-ERIC Directory (International Society for Biological and Environmental Repositories, 2022). ISBER offers a comparable resource through its Biobank Assessment Tool, a nearly 300-item self-assessment that benchmarks a repository against ISBER Best Practices and, at a higher level, against ISO 20387:2018, and returns a personalized report of areas for improvement (BBMRI-ERIC, 2026b). Additional organizations offering training, consultancy, and quality management support relevant to ISO 20387:2018 readiness are summarized in Supplementary Table S2. An outside audit or consultancy support provides biobanks with an objective assessment of their readiness level, alongside the interdisciplinary workgroup’s own preparation.

### 4.3 Financial and sustainability considerations

Financial value, operational efficiency, and social acceptability are the three main areas where the implementation of ISO 20387:2018 has quantifiable benefits. A shorter implementation period enhances financial efficiency by reducing resource use. Previous literature estimates the financial commitment associated with ISO 20387:2018 accreditation at approximately EUR 15–25K, in addition to personnel time, training, and infrastructure investments (Brancato et al., 2024). By reusing ISO 9001:2015-aligned processes and focusing implementation on ISO 20387-specific gaps, the proposed roadmap is intended to concentrate resources on activities that add new value, thereby potentially shortening the active preparation period and lowering overall resource use. The estimated 8-week effort required from the interdisciplinary workgroup may further reduce costs compared to the 2–4 persons/months normally required over an 8–12-month period (De Blasio and Biunno, 2021). It has also been shown that integrating an ISO 9001:2015-aligned quality management system can lead to cost savings by minimizing expenses on equipment, infrastructure, validations, and training (Pereira et al., 2015; Laun et al., 2022; Hannigan et al., 2019; Toniolo et al., 2009).

In the global context, financial sustainability has become increasingly relevant, as the global biobank market is projected to grow at a compound annual growth rate of 4.6% between 2022 and 2030, driven by investments in regenerative and personalized medicine (Grand View Research, 2022). As demand and operational complexity increase, engaging stakeholders in strategic decision-making is essential to connect biobank operations with the research community, thereby enhancing usage rates and supporting long-term sustainability (Coppola et al., 2019; Klingler et al., 2022; De Souza, 2015; Albert et al., 2014; Sargsyan et al., 2015).

To ensure financial sustainability, ISO 20387:2018 provides a structured and business-oriented management approach that blends quality governance and financial planning. However, biobanks rarely recover their full operating costs through specimen distribution fees alone; realistic recovery rates range from 5% to 25% (Klingler et al., 2022). Financial planning under ISO 20387:2018 should draw on a comprehensive cost model, such as the Total Life Cycle Cost of Ownership framework (Vaught et al., 2011), rather than assuming that cost recovery alone will keep biobanks solvent.

Beyond the financial perspective, ISO 20387:2018 enhances operational efficiency, standardizes processes, and improves the reliability and consistency of stored BMaD (Doucet et al., 2017). Standardized protocols also improve biobank interoperability by facilitating the integration and exchange of high-quality data. The advancement of thorough, comprehensive disease research and the development of data-driven technologies that support the goals of precision medicine depend on this type of interoperability.

Meeting these operational needs requires highly trained professionals, including biobank managers and personnel, supported by graduate or postgraduate training (for example, MSc Biobanking from the Medical University of Graz and Master in Biobanks and Complex Data Management of Université Côte d’Azur). These programs provide the capacity to transform theoretical knowledge into practical applications, ensuring the successful execution of processes necessary to attain and maintain compliance with international standards (Sargsyan, 2023).

Adherence to high-quality standards may contribute to strengthening public confidence in research results, as the use of standardized and accredited biological specimens can support the reliability and validity of biobanking practices (De Blasio and Biunno, 2021).

Therefore, the accreditation may increasingly be considered by users, stakeholders, and researchers as an indicator of quality and reliability in biobanking. As accreditation frameworks such as ISO 20387:2018 become more widely adopted, they could potentially play a greater role in future funding and research requirements (Abdaljaleel et al., 2019).

### 4.4 Potential challenges and mitigation strategy

By consolidating crosswalk findings with published cost and sustainability data, the roadmap outlines how a structured implementation process may support social acceptability, financial value, and operational efficiency. These benefits require empirical evaluation in future multicenter implementation studies. However, its implementation may be influenced by substantial organizational, financial, human resource, technical, and regulatory constraints. These challenges are particularly relevant for small biobanks (e.g., hospital- or university-based) that operate with limited personnel, shared institutional resources, early quality management systems, and restricted access to dedicated technical or administrative support (See Table 2).

**TABLE 2:** Potential implementation challenges and mitigation strategies for the proposed ISO 20387:2018 implementation roadmap.

Challenge	Potential impact on implementation	Possible mitigation/adaptation strategy
Financial constraints	May limit the ability to fund personnel time, training, consultancy, IT systems, documentation, data storage, quality management activities, infrastructure, and conformity assessment.	Implement the roadmap in phases, prioritize critical gaps, distinguish one-time implementation costs from recurring operational costs, incorporated into the five-to-ten-year financial plan, and identify diversified funding sources.
Limited personnel availability	Staff may have insufficient time to participate in the implementation workgroup or complete additional documentation, training, and quality-management activities.	Combine responsibilities where appropriate, designate an implementation coordinator, allocate protected time for implementation activities, provide targeted training, and use shared institutional or external expertise when required.
Immature or absent Quality Management System	May increase implementation time and require substantial foundational work before addressing ISO 20387:2018- specific requirements.	Introduce a preliminary QMS-development phase covering document control, SOPs, personnel training and competency, equipment management, risk management, internal audits, corrective actions, and management review before the ISO 20387:2018 gap analysis and implementation activities.
Limited institutional support and resistance to change	May hinder resource allocation, assignment of responsibilities, cross-departmental coordination, and adoption of standardized processes. Staff may perceive implementation as additional administrative burden or be reluctant to modify established practices.	Obtain early management endorsement, engage institutional leadership and key stakeholders, clearly define responsibilities, provide targeted training and awareness activities, communicate the strategic and operational benefits of accreditation, integrate implementation objectives into institutional planning, and introduce changes progressively.
Limited Information Technology infrastructure	May compromise traceability, data management, access control, data integrity, backup, and long-term preservation of biological material-associated data.	Assess existing IT systems against applicable ISO 20387:2018 requirements, prioritize critical gaps, leverage existing institutional infrastructure where appropriate, and implement technological improvements incrementally to the biobank’s scope and complexity.
Lack of specialized expertise	May limit the ability to interpret ISO 20387:2018 requirements or implement specialized technical, quality, regulatory, or data-management processes.	Provide target training, use external consultancy, mentoring, or institutional expertise when required, and leverage available biobanking assessment tools and quality-management resources.
National and regional regulatory differences	May introduce additional or differing requirements related to legislation, ethics, privacy, informed consent, data protection, sample transfer, ownership, and long-term storage.	Include applicable national and regional legislation and ethical requirements in the initial gap analysis, involve legal and ethics expertise when necessary, and maintain regulatory requirements register that is periodically reviewed and updated.
Organization size and operational complexity	May affect the number of personnel and departments involved, coordination requirements, decision-making processes, and the feasibility of the proposed multidisciplinary implementation structure.	Establish clear governance and decision-making structures, define responsibilities using a responsibility matrix, adapt the size of the implementation team and workstreams to the biobank’s scope, resources, processes, and operational complexity while ensuring that all required competencies and responsibilities are covered.

Since biobanks vary substantially in organizational structure, resource availability, QMS experience, regulatory context, and operational complexity, the proposed implementation roadmap should be adapted to each organization’s specific characteristics and readiness. This need for adaptation is also reflected in the BBMRI-ERIC quality-management framework, which recognizes that not all biobanks have sufficient resources to obtain certification from an accredited body. Instead, it provides stepwise quality-management support including self-assessment, audits, training, and other quality improvement services (BBMRI-ERIC, 2026b). Similarly, the BBRMI.be B3-ISO initiative provides implementation guidance and resources addressing quality management, information technology, ethical and legal issues, data management, traceability, and sustainability (BBMRI.be, 2025).

### 4.5 Limitations

First, the roadmap and timeline are based on a single organizational setting with an established ISO 9001:2015-certified QMS and may not be directly generalizable to other contexts. Second, the guide is specifically proposed for biobanks already operating under ISO 9001:2015; institutions without such a system will require additional foundational work. Third, the estimated 17–20-week implementation timeframe should be regarded as an approximation rather than a universal benchmark, as implementation speed will vary with organizational maturity, resources, and prior experience. Together, these limitations underscore the need for multicenter implementation studies to validate and refine the proposed roadmap.

## 5 Conclusion

By combining a clause-level crosswalk between ISO 9001:2015 and ISO 20387:2018 with an organizational implementation example, this study provides a structured, role-based roadmap for ISO 20387:2018 readiness in ISO 9001:2015-certified biobanks. Future multicenter implementation studies should evaluate the feasibility, resource requirements, and impact of this roadmap in diverse biobank settings, including institutions without pre-existing ISO 9001:2015 certification.

## Data Availability

The original contributions presented in the study are included in the article/Supplementary Material, further inquiries can be directed to the corresponding author.
