# Supplementary material for: From ISO 9001:2015 to ISO 20387:2018: a proposed implementation roadmap for biobanks
Source: Front Genet. 2026 Sep 30;17:1953941. doi: 10.3389/fgene.2026.1953941 (PMC13661020; doi:10.3389/fgene.2026.1953941)
Supplement: Supplementary file 1 [file Table1.docx]

**Supplementary Table S1.** Clause-by-clause crosswalk between ISO 20387:2018 and ISO 9001:2015

| **ISO 20387:2018 Clause** | **Requirement / Topic** | **Corresponding ISO 9001:2015 clause(s)** | **Overall correspondence with ISO 9001:2015** |
| --- | --- | --- | --- |
| 4 | General Requirements |  |  |
| 4.1 | General | 4.1, 4.2, 4.3, 4.4, 5.1.2, 5.3, 6.1, 6.2, 7.1.6, 7.2, 7.5, 8.1, 8.2.3, 8.5 | YES |
| 4.2 | Impartiality | 4.1, 5.2, 5.3, 6.1 | YES |
| 4.3 | Confidentiality | 5.3, 7.2, 7.5.3, 8.2, 8.5.3 | YES |
| 5 | Structural Requirements |  |  |
| 5.1 | Be a legal entity, or a defined part of a legal entity | 4.1, 4.2 | YES |
| 5.2 | Identify top management | 5.1.1, 5.3 | YES |
| 5.3 | Governance body/advisory board | 5.3 | YES |
| 5.4 | Responsible for activities | 5.3 | YES |
| 5.5 | Define and address liabilities arising from its activities | 7.5 | YES |
| 5.6 | Comply with legal norms and agreements | 7.5.3, 8.2.3, 8.5.3 | YES |
| 5.7 | Define and document the range of activities | 7.5, 8.4 | YES |
| 5.8 | Define the governance structure and requirements of personnel | 4.1, 5.3, 7.1.2, 7.2, 7.5 | YES |
| 5.9 | Personnel | 5.3, 7.2, 7.3, 8.7, 9.1 | YES |
| 5.10 | Communication | 7.4, 8.2.4, 8.4.3, 8.5.6, 8.7, 9.1 | YES |
| 6 | Resource requirements |  |  |
| 6.1 | General | 7.1, ---- | PARCIAL |
| 6.2 | Personnel | 5.3, 6.1, 7.1.4, 7.2, 7.3, 7.4 | YES |
| 6.3 | Facilities/dedicated areas and environmental conditions | 6.1, 7.1, ---- | PARCIAL |
| 6.4 | Externally provided processes, products and services | 6.1, 7.4, 8.4, 9.2 | YES |
| 6.5 | Equipment | 6.1, 7.1.5, 7.5.3, 8.1, 8.5.1, 8.7 | YES |
| 7 | Process requirements |  |  |
| 7.1 | General | 7.5, 8.1 | YES |
| 7.2 | Collection of biological material and associated data | 5.3, 8.1, 8.2, ---- | PARCIAL |
| 7.3 | Reception and distribution of biological material and associated data | 8.1, 8.2, 8.4, 8.5.1, 8.7, ---- | PARCIAL |
| 7.4 | Transport of biological material and associated data | 5.3, 7.1.2, 7.1.3, 7.1.4, 8.1, 8.2, 8.5.1, 8.7 | YES |
| 7.5 | Traceability of biological material and associated data | 5.3, 7.1.2, 7.1.3, 7.5, 8.1, 8.5.1 | YES |
| 7.6 | Preparation and preservation of biological material | 7.5, 8.1, 8.2 | YES |
| 7.7 | Storage of biological material | 6.1, 7.1, 8.1, 8.5 | YES |
| 7.8 | Quality control of biological material and associated data | 8.1, 8.5, 8.6, 9.1, 10 | YES |
| 7.9 | Validation and verification of methods | 7.5, 8.1, 8.2.3, 8.5.1, 8.5.6 | YES |
| 7.10 | Management of information and data | 7.1.3, 7.5, 8.1, 8.2, 8.5.3, 8.6, ---- | PARCIAL |
| 7.11 | Nonconforming output | 8.2.1, 8.7 | YES |
| 7.12 | Report requirements | 8.1, 8.2.1, ---- | PARCIAL |
| 7.13 | Complaints | 10.2 | YES |
| 8 | Quality management system requirements | ISO 9001:2015 | YES |

This table provides a summarized representation of the complete clause-by-clause crosswalk. Because multiple subclauses may be grouped under a single ISO 20387:2018 clause in the summary table, the “Addressed by ISO 9001:2015” column indicates the overall degree of correspondence within each grouped clause. “Yes” indicates that all assessed requirements within the summarized clause were addressed by corresponding ISO 9001:2015 requirements; “Partial” indicates that some, but not all, requirements within the summarized clause were addressed.

**Supplementary Table S2.** International Organizations and Resources supporting Quality Management and ISO 20387 implementation in Biobanking.

| **Organization** | **Resources/Services Offered** | **Intended Users** | **Website** |
| --- | --- | --- | --- |
| BBMRI-ERIC  (Quality Management Services) | Knowledge hub, training & support on standards (including ISO 20387), self-assessment surveys, remote/on-site audits, Quality Label / Q-Mark, consultancy for QMS implementation, and continuous improvement guidance. | Biobanks, laboratories, and researchers (primarily in BBMRI-ERIC member states; some services more widely available). | <https://www.bbmri-eric.eu/services/quality-management/> |
| ISBER | ISBER Best Practices for Repositories (5th Edition) – global guidance on quality management, operations, ethics, and specimen handling. Education, webinars, working groups, and tools that support QMS development and ISO 20387 readiness. | Biobank professionals, managers, and organizations worldwide (human, animal, plant, environmental repositories). | [<https://www.isber.org/page/BPR>](https://www.isber.org/page/BAT) |
| ESBB | Scientific networking, training/education, working groups (including Quality Management, Audits & Accreditation), conferences, and promotion of high professional standards and harmonization. | Biobank community across Europe, Middle East, and Africa (and beyond). |  |
| Medical University of Graz / Biobank Graz (International Biobanking Education**)** | English-language training program (Basic: “How to build a biobank”; Advanced: “How to Operate a Biobank”; Sustainability in Biobanking), distance-learning MSc in Biobanking, and individual training/support on QMS, ISO/CEN standards, sample workflows, ethics, IT, sustainability, and strategy. | International biobankers, staff setting up or improving biobanks, and related professionals. | <https://www.medunigraz.at/en/international-biobanking-education> |
| U.S. National Cancer Institute  (NCI Best Practices for Biospecimen Resources) | Detailed governance, ethical, operational, and technical best practices for biospecimen resources. Supports quality systems and are often used alongside ISO 20387 or CAP. | NCI-supported and other biospecimen resources, especially cancer-focused biobanks (widely referenced globally). | [Best Practices for Biospecimen Resources - NCI](https://dctd.cancer.gov/data-tools-biospecimens/biospecimens-biobanks/resources/best-practices/biospecimen-resources) |

**Abbreviations:** BBMRI-ERIC, Biobanking and BioMolecular Resources Research Infrastructure–European Research Infrastructure Consortium; CAP, College of American Pathologists; ESBB, European, Middle Eastern & African Society for Biopreservation and Biobanking; ISBER, International Society for Biological and Environmental Repositories; ISO, International Organization for Standardization; QMS, Quality Management System.
